# RETRACTION: Effects of Clinical Nursing Pathway on the Surgical Site Wound Infection in Patients Undergoing Knee or Hip Replacement Surgery: A Meta‐Analysis

**DOI:** 10.1111/iwj.70390

**Published:** 2025-03-21

## Abstract

**RETRACTION:** HuangZ. , LiY. , PengJ. , WangH. , ShenK. , LiY. , and YuK. , “Effects of Clinical Nursing Pathway on the Surgical Site Wound Infection in Patients Undergoing Knee or Hip Replacement Surgery: A Meta‐Analysis,” International Wound Journal 21, no. 3 (2024): e14657, 10.1111/iwj.14657.38472128 PMC10932801

The above article, published online on 12 March 2024, in Wiley Online Library (http://onlinelibrary.wiley.com/), has been retracted by agreement between the journal Editor in Chief, Professor Keith Harding; and John Wiley & Sons Ltd. Following an investigation by the publisher, all parties have concluded that this article was accepted solely on the basis of a compromised peer review process. The editors have therefore decided to retract the article. The authors disagree with the retraction.

**RETRACTION:** Z. Huang , Y. Li , J. Peng , H. Wang , K. Shen , Y. Li , and K. Yu , “Effects of Clinical Nursing Pathway on the Surgical Site Wound Infection in Patients Undergoing Knee or Hip Replacement Surgery: A Meta‐Analysis,” International Wound Journal 21, no. 3 (2024): e14657, 10.1111/iwj.14657.38472128 PMC10932801

The above article, published online on 12 March 2024, in Wiley Online Library (http://onlinelibrary.wiley.com/), has been retracted by agreement between the journal Editor in Chief, Professor Keith Harding; and John Wiley & Sons Ltd. Following an investigation by the publisher, all parties have concluded that this article was accepted solely on the basis of a compromised peer review process. The editors have therefore decided to retract the article. The authors disagree with the retraction.

